# A Review of the Effects of Valenced Odors on Face Perception and Evaluation

**DOI:** 10.1177/20416695211009552

**Published:** 2021-04-28

**Authors:** Elmeri Syrjänen, Håkan Fischer, Marco Tullio Liuzza, Torun Lindholm, Jonas K. Olofsson

**Affiliations:** Department of Psychology, Stockholm University, Stockholm, Sweden; Department of Psychology, Stockholm University, Stockholm, Sweden; Department of Surgical and Medical Sciences, “Magna Graecia” University of Catanzaro, Catanzaro, Italy; Department of Psychology, Stockholm University, Stockholm, Sweden

**Keywords:** review, multisensory/cross-modal processing, odor, face perception

## Abstract

How do valenced odors affect the perception and evaluation of facial expressions? We reviewed 25 studies published from 1989 to 2020 on cross-modal behavioral effects of odors on the perception of faces. The results indicate that odors may influence facial evaluations and classifications in several ways. Faces are rated as more arousing during simultaneous odor exposure, and the rated valence of faces is affected in the direction of the odor valence. For facial classification tasks, in general, valenced odors, whether pleasant or unpleasant, decrease facial emotion classification speed. The evidence for valence congruency effects was inconsistent. Some studies found that exposure to a valenced odor facilitates the processing of a similarly valenced facial expression. The results for facial evaluation were mirrored in classical conditioning studies, as faces conditioned with valenced odors were rated in the direction of the odor valence. However, the evidence of odor effects was inconsistent when the task was to classify faces. Furthermore, using a *z*-curve analysis, we found clear evidence for publication bias. Our recommendations for future research include greater consideration of individual differences in sensation and cognition, individual differences (e.g., differences in odor sensitivity related to age, gender, or culture), establishing standardized experimental assessments and stimuli, larger study samples, and embracing open research practices.

## Introduction

Inferring other peoples’ emotional states from their facial expressions is an essential social ability. Historically, facial perception has been studied in isolation; however, social cues are often ambiguous and rely heavily on contextual information for correct interpretation (Aviezer et al., 2017). How we interpret facial expressions may vary by previous experiences, cultural background, and context (Barrett et al., 2019). In line with this reasoning, several recent reviews have considered the effects of auditory context (Gerdes et al., 2014), visual cue context, background scene, body posture, situation, culture, and individual differences (Aviezer et al., 2017; Wieser & Brosch, 2012). However, the contextual effects of odors on facial perception have received less interest. People are normally capable of detecting environmental odors even at minute concentrations (McGann, 2017), but olfactory perception needs visual confirmation to establish its identity or social relevance (Olofsson & Gottfried, 2015). This research review aims to integrate the emerging insights from how odors are perceptually integrated with emotionally expressive face stimuli.

Olfaction provides an interesting vantage point for studying cross-sensory integration of emotional cues. Olfaction has been described as an inherently emotionally evocative sense and is well integrated with the limbic system (Soudry et al., 2011). Olfaction is involved in detecting and identifying health hazards (e.g., fire, bad food) and food and nutrition (de Vries et al., 2020; Stevenson, 2010). This link between olfaction and survival might explain the emotional nature of the olfactory system. For example, valenced odors can elicit basic emotions, most notably happiness and disgust (Alaoui-Ismaïli et al., 1997; Croy et al., 2011; Glass et al., 2014). Thus, odors have profound emotional and motivational effects on human behavior.

Due to the close link between odors and emotions, odors might affect the processing of social cues. There is a broad agreement that olfaction’s main hedonic dimensions are valence and arousal (Anderson et al., 2003; Bensafi, 2002). Valenced odors might affect mood states such that pleasant odors, unsurprisingly, have a positive effect. In contrast, unpleasant odors have a negative effect on mood (Herz, 2009), and odors may also be experienced as energizing, sensual, or disgusting (Ferdenzi et al., 2011). In general, the arousing properties of odors may facilitate the overall processing of stimuli. Corroborating this notion, Millot et al. (2002) found faster response times (RTs) to simple visual and auditory cues in both unpleasant and pleasant odor contexts compared with a no-odor context. Apart from the general arousing properties of odors, there is evidence of odor-visual integration from research on object congruency. Smelling an orange can increase visual attention toward oranges (Seo et al., 2010). Behavioral studies also show that congruent cues from olfactory and visual objects are processed in parallel in the early stages of stimulus processing, but they are combined at later stages to benefit behavioral decisions (Amsellem et al., 2018). Thus, there are several ways by which odors might influence facial perception, including arousal and valence effects, as well as their interactions with facial expressions that are congruent or incongruent with the emotion elicited by the odor.

In ecological settings, perception is often multisensory. Previous research has shown that when we use congruent cues from multiple modalities, our ability to recognize objects becomes faster and more accurate. For example, in a noisy environment, seeing a speaker’s lips will improve the ability to hear the verbal content (Ross et al., 2007). Odors can, in a similar manner as hearing, influence visual perception. Most research on the influence of odors on perception has focused on matching odors and visual object cues (e.g., the smell and picture of an orange), odor-visual stimuli that have a natural correspondence (Olofsson et al., 2014, 2018). Here, we focus instead on the relation between odors and perception of facial expressions. Arguably, the association between an unpleasant odor and a disgusted face is just as natural as the connection between the smell of an orange and an orange. Humans commonly react to and express their liking and disliking of the olfactory environment with facial expressions (Majid et al., 2018). Effectively integrating odor and face cues might help evaluate the source and potential health hazards associated with the odor. However, the emotional properties of odors might also lead to biased evaluations of face stimuli. The current review thus focuses on how odors affect the perception of faces.

The basic emotion theory (Ekman et al., 1969) posits six universal basic emotions (happiness, fear, disgust, anger, surprise, and sadness), each associated with distinct facial expressions, physiological changes, and behavioral patterns. However, many of these assumptions, such as the universality (Russell, 1993), facial expressions (Barrett et al., 2019), and physiological changes (Lindquist et al., 2012) of the basic emotion theory have been questioned. As noted earlier, recent theoretical work has emphasized the contextual effects in emotions (Barrett, 2017a). In this view, in emotion perception, all available information is used to predict and correct our model of the world using active inference such as past experiences, culturally appropriate displays, and contextual information in our environment (Barrett, 2017b). The last three decades have seen a steady increase in research on contextual effects on facial perception. In our current review, we retain the classification of emotional expressions provided by the basic emotion theory. However, we highlight the importance of how an olfactory context affects facial perception and associated behaviors. The behavioral measures comprise affective ratings, emotion classification accuracy, and RTs in various tasks. Furthermore, to investigate publication bias (i.e., selective reporting of only statistically significant results), we used *z*-curve 2.0 (Bartoš & Schimmack, 2021) to estimate expected discovery rate (EDR) and expected replication rate (ERR).

## Method

We performed searches in Google Scholar, PsycINFO, PubMed, and Web of Science for keywords (face, odor, smell, olfaction, and cross-modal), as well as in our databases of articles, with the last search conducted on September 1, 2020. Articles matching our criteria were also manually searched in the reference lists of all included articles. We did not include studies attempting to study human pheromones or chemosignals. As this research is based on assumptions of unique properties of certain chemosensory stimuli, these reports fell outside of our review’s scope to integrate research findings across different stimuli and experimental settings whenever possible. We included articles that fulfilled the following inclusion criteria.

1. Peer-reviewed empirical studies available in English
2. Healthy adult participants
3. Reported behavioral effects of odors on the perception of faces

### Z-Curve

We used the *z*-curve package in R to conduct *z*-curve analyses (Bartoš & Schimmack, 2021). The *z*-curve method is designed to estimate publication bias by using observed *p*-value or *z*-scores in published studies. The *z*-curve method attempts to estimate the conditional mean power by using all the significant results in the published studies. Using this estimate of mean power, it is possible to calculate the ERR, that is, the success rate if these studies would be exactly replicated. The *z*-curve 2.0 method includes a feature to calculate the unconditional mean power, that is, an estimate of the power in studies that never were published because of statistically nonsignificant findings. Publication bias can be calculated from the unconditional mean power, that is, if the EDR (an estimate of the proportion of statistically significant results in all conducted studies, both published and unpublished) is lower than the observed discovery rate (ODR; i.e., statistically significant results in published studies). Using the EDR, we can also estimate the proportion of false positive findings in the literature. Detailed descriptions of these methods are published elsewhere (see; Bartoš & Schimmack, 2021; Brunner & Schimmack, 2020).

Of the 25 published studies, 24 contained test-statistics or exact *p*-values that could be analyzed with the *z*-curve method. From these studies, we manually extracted test statistics that were relevant for odor effects on face perception. Where applicable, we extracted statistics for the full analysis of variance and not follow-up contrast analyses. We also computed EDR and ERR on a random sample of *p*-values from each study because the *z*-curve has only been validated on test statistics in independent studies (i.e., one focal test per study). Specifically, we ran the *z*-curve analysis for 500 iterations and randomly sampled one *p*-value from each independent experiment (*n* = 27) in the studies with enough information to perform the *z*-curve analysis (*n* = 24).

## Results

In total, we found 25 articles matching the inclusion criteria; these articles were published from 1989 until September 2020 and are presented with summaries in Table 1. The table comprises the studies listed in chronological order, an overall description of the design, stimuli, participants, and main findings.

**Table 1.** Description and Main Results of Behavioral Studies of Face Perception During Odor Exposure, Studies Listed Chronologically.

Study (year)	Design	Paradigm Classification/Evaluation/Conditioning	Stimuli Odor/face (O/F)	Sample size Total sample (female)	Main findings
Cann & Ross (1989)	Attractiveness of female faces during exposure to ple, no-odor, or unp odors.	Evaluation	O: Ple, no-odor, unpF: Neu	63 M	Odors did not affect attractiveness ratings.
Todrank et al. (1995)	Liking of neu faces paired with ple, neu, or unp odors. Exp 1, 2, 4 people-related odors. Exp 3, object-related odors.	Evaluation	O: Ple, neu, unpF: Neu	Exp 1: 20 (15 F) Exp 2: 15 (11 F) Exp 3: 14 (9 F) Exp 4: 23 (16 F)	Faces paired with people-related odors were liked in the direction of odor valence in Exp 1,2 & 4. This effect was not evident in Exp 3, where object-related odors were used.
Hermann et al. (2000)	Val and arousal ratings (SAM-scale) of neu faces conditioned by startle sounds during exposure to ple (*n* = 15) or unp odor (*n* = 15).	Conditioning	O: Ple, unp F: Neu	30 M	Faces paired with a startle sound during unp odor exposure were rated as more arousing and neg than other conditions. Faces paired with a startle sound during a ple odor exposure were rated as more arousing but not more pos.
Bensafi, Pierson, et al. (2002)	Neutral faces evaluated and classified as ple or unp during exposure to ple odor or no-odor.	Evaluation & classification	O: Ple, no-odor F: Neu	15 F	Plea odor did not affect face evaluations nor classification.
Gottfried et al. (2002)	Neu faces paired with ple, no-odor, or unp odor. RTs obtained in face gender classification task.	Conditioning	O: Ple, no-odor, unp F: Neu	17 (10 F)	Slower RTs for faces conditioned to ple and unp odors in the first block, no-effect in the second and third block.
Leppanen & Hietanen (2003)	Exp 1. Face emotion classification task (happy or disgusted) during ple or unp odor exposure. Exp 2. Similar to 1 but with a between-groups design, added neu faces, and no-odor controls.	Classification	Exp 1. O: Ple, unp F: Happy, DisgustedExp 2.O: Ple, no-odor, unpF: Happy, neu, disgusted	Exp 1: 20 (15 F) Exp 2: 45 (40 F)	Exp 1. Happy faces recognized faster in ple odor context; effect was absent in unp odor condition, where disgusted faces were recognized slightly faster. Exp 2. Happy faces recognized faster in ple odor context, slower in no-odor control, and slowest in unp odor condition.
Gottfried & Dolan (2004)	2 CS+ neutral faces paired with UCS odors, 2 faces CS− not paired with an unp odor. RTs for gender identification, faces were rated (VAS) on intensity and valence.	Conditioning & Evaluation	O: Unp F: Neu	18 (10 F)	Faster RTs to CS+ first half of the exp. CS+ faces were rated as more neg val compared with CS−.
Dematte et al. (2007)	Women rated (VAS) attractiveness of male faces during exposure to ple and unp odors.	Evaluation	O: Ple, unp F: Neu (varying in attractiveness)	16 F	Faces rated as less attractive during exposure to unp odor.
W. Li et al. (2007)	Likeability of neu faces rated (VAS) after exposure to sub-threshold ple, neu, no-odor, and unp odors.	Evaluation	O: Ple, neu, no-odor, unp F: Neu	31 (18 F)	Odor-unaware subgroup (*n* = 15) rated faces as less likable after unp than after ple odor.
Seubert, Loughead, et al. (2010)	Emotion classification task (happy, neu, disgusted faces) in event-related design with ple, no-odor, and unp odors.	Classification	O: Ple, no-odor, unp F: Happy, neu, disgusted	24 (10 F)	Faster and more accurate classification of disgust in ple and unp odor condition than in neutral odor condition. Response times to happy faces increased in ple and unp odor conditions compared with neu odor.
Seubert, Kellermann, et al. (2010)	Emotion classification task (on happy, neu, and disgusted faces) in an event-related design with a ple, no-odor, and unp odors.	Classification	O: Ple, no-odor, unp F: Happy, neu, disgusted	44 (23 F)	Faster classification of disgust in ple and unp odor.
Steinberg et al. (2012)	Face valence and arousal ratings (SAM) to CS+ (unp odor) and CS− faces.	Conditioning & evaluation	O: Unp, no-odor F: Neu	24 (12 F)	CS+ faces rated as more negative post conditioning, no arousal effects.
Steinberg et al. (2013)	Preference for neu faces when previously paired with ple, no-odor, or unp odors.	Conditioning & evaluation	O: Ple, no-odor, unp F: Neu	23 (12 F)	Preference for face depended on associated odor (pleasant > neutral > unpleasant).
Seubert et al. (2014)	Classification of target faces as older or younger than a cue face. Ratings (VAS) of age and attractiveness.	Evaluation	O: Ple, unp odor mixtures controlling for intensity (linearly increasing in val from ple to unp). F: Neu (varying in attractiveness and age)	18 (12 F)	Faces were rated as more attractive and younger in ple compared with unp odor condition.
Cook et al. (2015)	Val ratings (VAS) of neu faces primed by a ple, no-odor, or an unp odor.	Evaluation	O: Ple, no-odor, unp F: Neu	23 (12 F)	Ratings of faces were affected by odor priming in the direction of odor val.
Leleu et al. (2015)	Matching faces with varying levels of emotional expressions during exposure to ple, no-odor, and unp odor.	Classification	O: Ple, no-odor, unp F: Happy, angry, fearful & sad (morphed with neu for emotion levels)	48 (31 F)	Congruent odors increased sensitivity for face emotion matching (e.g., happy faces in the ple odor condition) for happy, disgusted, and angry expressions. Fewer false alarms for disgusted faces in the unp odor condition when emotion labels for faces were provided. Without labels, more false alarms were provided for neg emotions in the ple odor context.
Novak et al. (2015)	Val ratings (VAS) and emotion classification of neu and fearful faces during neu or sub-threshold unp odor exposure.	Evaluation & classification	O: Neu, unp (diluted with neu odors below detection threshold) F: Neu (morphed with fearful faces in 2% or 12%)	16 (8 F)	No effects of odors on rated val or emotion recognition accuracy.
Cook et al. (2017)	Val ratings (VAS) of neutral faces during exposure to a ple, unp, or neu odor.	Evaluation	O: Ple, no-odor, unp F: Happy, disgusted	23 (13 F)	Happy faces were rated more ple during exposure to a ple odor than neu or unp odor; disgusted faces rated as less ple during exposure to unp odor compared with plea and neu odor.
Syrjänen et al. (2017)	Ratings (VAS) of facial expressions during exposure to unp, no-odor, and ple conditions. RTs and accuracy in emotion classification task of changing faces.	Evaluation & classification	O: Ple, no-odor, unp F: Happy, neu, disgusted	21 (13 F)	Odors did not affect ratings of face valence or arousal. Faster classification of faces in unp odor condition. Classification accuracy was not affected by odors.
Damjanovic et al. (2018)	Detection of facial emotion in a visual search task during odor exposure (ple, no-odor, or unp odors).	Classification	O: Ple, no-odor, unp F: Happy, neu, angry	54 (43 F)	Faster detection of happy faces in neu and unp odor conditions. Similar effect for ple odor early in the experiment, but effect decreased over time. In contrast, unp odor made detection of happy faces faster later in the experiment.
Cook et al. (2018)	Valence ratings of neutral faces during, or one second after exposure to a ple, no-odor, or unp odor.	Evaluation	O: Ple, no-odor, unp F: Neu	28 (18 F)	Faces rated less ple when primed with unp odor; effect largest when odor and faces were presented concurrently.
Syrjänen et al. (2018)	Val and arousal ratings (SAM) of facial expressions during exposure to unp, ple, and no-odor	Evaluation	O: Ple, no-odor, unp F: Happy, neu, disgusted	58 (33 female)	Faces rated more neg during unp odor exposure. Happy faces were rated as less arousing in unp than ple odor condition.
Syrjänen et al. (2019)	Val and arousal ratings (SAM) of facial expressions during exposure to unp, ple, and no-odor	Evaluation	O: Ple, no-odor, unp F: Happy, neu, disgusted	40 (22 F)	More pos ratings in the ple odor and more neg ratings in the unp odor exposure. Higher arousal ratings during odor exposure vs. no-odor.
D. Li et al. (2020)	Emotion classification task on happy, neu, and fearful faces during exposure to ple, neu, or unp odors.	Classification	O: Ple, no-odor, unp F: Happy, neu, fearful	54 (28 F)	Higher accuracy during unp odor exposure. Faster classification in unp odor condition. Effect was pronounced for fearful faces.
Stankovic et al. (2020)	Binary face classification task (emotion vs. neutral) with stimulus in right or left visual field. Breathing through either left or right nostril during unp odor exposure.	Classification	O: Unp F: Neu, happy, surprised, fearful, angry, disgusted, sad	60 F	Better classification of emotional faces in the right than the left visual field during unp odor exposure. Participants faster at recognizing faces in left visual field during exposure to odors in right nostril.

*Note.* Unp = unpleasant; ple = pleasant; neu = neutral; pos = positive/positively; neg = negative/negatively; exp = experiment; val = valence/valenced; SAM = self-assessment manikin; UCS = unconditioned stimulus; CS = conditioned stimulus; RT = response time; VAS = visual analog scale.

An overview of the included studies allows for some general observations regarding the most commonly used methods and stimuli. The methods can be roughly divided into three experimental paradigms. First, facial emotion classification tests where RT and accuracy levels were measured. Second, subjective evaluations (e.g., ratings of facial valence and arousal) during exposure to various odors. Third, classical conditioning studies where neutral faces were associated with valenced odors; these studies used both emotion classification tasks and facial evaluations as outcome measures. Several studies included electrophysiological measures or functional magnetic resonance imaging; however, here, we focus on behavioral effects. Odor stimuli were typically either clearly pleasant (vanillin and floral-based, such as jasmine) or unpleasant (valeric acid, hydrosulfide, methyl mercaptan, and fish). A majority of the included studies also used clean air as a no-odor control condition; however, few studies used emotionally neutral odors as a control. Common facial expressions used in the reviewed studies were happy, neutral (most prevalent in associative learning studies), disgusted, fearful/anxious, and angry expressions.

**Figure 1.**
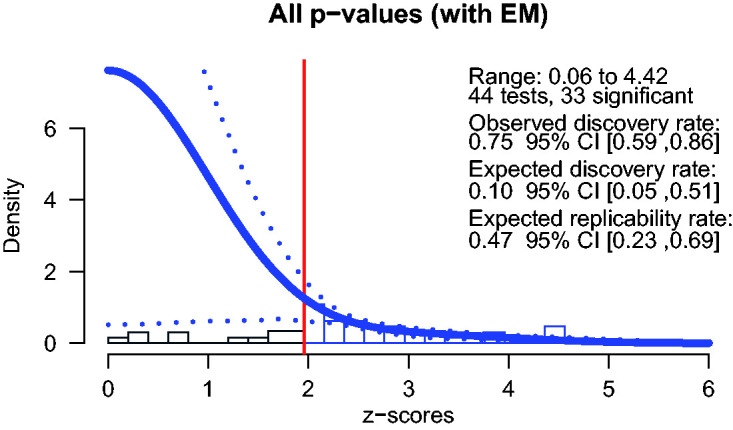
Estimated *z*-curve with the EM method for all *p*-values. The histogram shows the observed *z*-statistics in the included studies. The red vertical line shows the significance threshold at alpha .05 corresponding to a *z*-score of 1.96. The blue line denotes the density of the estimated model with 95% confidence lines shown with blue dotted lines. EM = expectancy-maximization; CI = confidence interval.

We also used *z*-curve 2.0 to investigate publication bias (Bartoš & Schimmack, 2021; Brunner & Schimmack, 2020). The ODR was .75 confidence interval (CI) [.59, .86]. The results of the *z*-curve analysis indicated that given the statistically significant results, the EDR was 0.10 CI [.05, .51], as the lower CI includes .05 we cannot reject the possibility that all results were false positives. However, the expected replicability rate .47 CI [.23, .69] indicates that there are true positive results among the studies. A visual inspection of the obtained results confirms these results (see Figure 1), there is a steep drop from just statistically significant values compared with nonsignificant values, suggesting publication bias.

**Figure 2.**
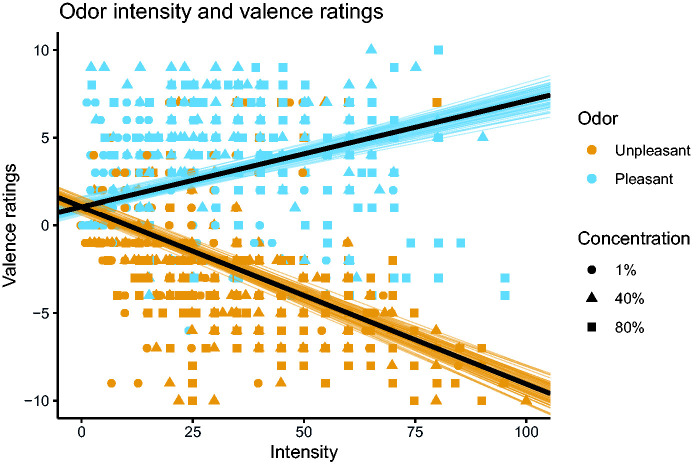
Rated odor intensity and valence for three different concentrations of valeric acid (unpleasant) and lilac essence (pleasant). The black lines show the predicted linear relationship between intensity and valence. Uncertainty of the linear effect (colored lines) is visualized with a sample of draws from the posterior distribution. Data from Syrjänen et al. (2018). Figure reprinted with permission from the author.

Hitherto, the *z*-curve method has been validated on independent studies on focal statistical tests. To account for nonindependence (i.e., most of the studies in this review contained multiple tests), we repeatedly drew a random sample of one *p*-value from each study and computed the, ODR, EDR, and ERR. The results were similar to the full analysis using all *p*-values, with an ODR of .78 CI [.74, .81], an EDR of .23 CI [.05, .41], and an ERR of .49 CI [.35, .66].

## Discussion

The overall pattern emerging from the reviewed studies suggests that valenced odors affect face evaluations. The most consistent finding is that valenced odors affect evaluations in general (e.g., all faces are perceived as more unpleasant in an unpleasant odor condition). This indicates that a valenced odor might change the participants’ emotional state, which biases their evaluation of faces, or that when making valence evaluations of a face, participants misattribute the odor valence to the face being evaluated. A few studies show odor-face congruency effects on evaluations (i.e., that an odor eliciting an emotion affects the processing of faces expressing specifically that emotion). In emotion classification tasks, a few of the studies found evidence for odor and facial expression integration. However, the majority of the findings indicated enhanced response latencies in odor conditions relative to no-odor control conditions. We also used *z*-curve to estimate publication bias. The results indicated that when considering the full body of work, studies had low power to detect statistically significant effects. We broadly classified the experimental paradigms into three categories, classification, evaluation, and classical conditioning. The results are reviewed in more detail later.

### Odor Effects on the Processing of Face Stimuli

In this review, nine studies used response times as a measure to investigate the effects of valenced odors on emotion recognition. Leppanen and Hietanen (2003) were the first to show that pleasant odors facilitated the classification accuracy of happy faces. In contrast, the speed of classifying disgusted faces was not affected by either pleasant or unpleasant odors. In the same study, a second experiment confirmed the effect of pleasant odors on happy faces. Although several studies have used similar methods as Leppanen and Hietanen (2003), their odor-face congruency effects have to our knowledge not yet been replicated. Instead, in a similar experimental design, D. Li et al. (2020) found that facial expressions (happy, neutral, and fearful) were overall recognized faster in an unpleasant food odor context. This effect was most pronounced for fearful faces. The effect was not present for happy faces in the pleasant odor condition. In two separate studies by Seubert et al., the results showed only an overall effect of odor presence on improving face classification (Seubert, Kellermann, et al., 2010; Seubert, Loughead, et al., 2010). In one of these studies, participants were more accurate and faster in recognizing disgusted expressions in both the pleasant and unpleasant odor conditions and slower at recognizing happy faces than in the neutral condition (Seubert, Kellermann, et al., 2010). In the second study, the results were similar, but this time only present for disgusted faces (Seubert, Loughead, et al., 2010). Hence, these results are difficult to consolidate. One reason for the heterogeneity of results might be that performance is already close to the ceiling level in a simple task such as emotion classification. If odor effects are generally relatively small, there is not much room for improvement. The results of Leleu et al. (2015) hint at this possibility. In this study, emotional expressions were morphed with neutral faces to show various levels of emotional intensity. The results demonstrated that facial expressions morphed with neutral faces were recognized at lower levels in congruent odor conditions (e.g., happy faces in the pleasant odor condition), for happy, disgusted, and angry facial expressions (Leleu et al., 2015). However, the results in a recent study (Syrjänen et al., 2017) showed that the recognition of dynamically morphing happy or disgusted faces (gradually emerging from neutral baseline face stimuli) was not affected by congruent odors. In the latter study, exploratory results indicated that both happy and disgusted expressions were recognized faster during exposure to an unpleasant odor (Syrjänen et al., 2017). Some studies have also presented stimuli in the left and right visual fields. In Stankovic et al. (2020), participants performed an emotion classification task on neutral or happy, surprised, fearful, angry, disgusted, and sad facial expressions. Pictures of faces were presented in the left or right visual fields while participants were exposed to an unpleasant odor in the left or right nostril. Participants were faster at recognizing faces overall in the left visual field (right hemisphere) during exposure to the odor in the right nostril (right hemisphere), indicating a cross-modal confluence in the right hemisphere resulted in a processing speed advantage; however, the study had no neutral control condition, so it is possible that the arousing effect of the odor, irrespective of its valence, improved processing of faces in the stimulated hemisphere (Stankovic et al., 2020). The right-hemisphere effect is consistent with results from our team that the face-sensitive N170 ERP component is more prominent in the right hemisphere; furthermore, our study showed that the responsivity of the N170 to disgusted faces is enhanced by an unpleasant odor (Syrjänen et al., 2018). Another recent study investigated whether odors might direct attention toward odor-congruent happy and disgusted facial expressions in a dot-probe task (Syrjänen et al., 2019). However, this study’s results gave no evidence that attention was automatically directed toward odor-congruent facial expressions. However, the results instead indicated that probes in the unpleasant odor condition were uniquely detected more rapidly over repeated trials within a block, raising the possibility that visual processing benefits of malodor exposure might accumulate over time. Similar time-on-task effects were found by Damjanovic et al. (2018) in a visual search task. The authors found that happy faces were recognized faster initially in a pleasant and an unpleasant odor context; however, this effect was reversed toward the end of the task.

While there is some evidence to suggest valenced odors might improve visual processing of emotionally congruent facial expressions, especially the combination of unpleasant odor and disgusted or fearful faces, the evidence is mixed. Increasing task difficulty, employing brief face duration times, subtle or morphed expressions, and so on, might be needed to achieve consistent effects of background odors. There are also some indications that odor effects develop as a function of exposure time during the experiment. Not accounting for such effects might to some extent explain why the findings are mixed.

### Odor Effects on Evaluations of Faces

Ten studies assessed how valenced odors affect the subjective properties of faces. Two of these studies showed that neutral faces were rated as more unpleasant in the context of an unpleasant odor (Cook et al., 2015, 2018) and more pleasant during a pleasant odor context (Cook et al., 2015). However, one study did not find any effect of pleasant odors on classifying neutral faces as either pleasant or unpleasant, which might indicate that binary classifications of faces are less sensitive than ratings to the presumably subtle influence of odors (Bensafi, Pierson, et al., 2002). Several studies have shown that faces, regardless of expression, are rated as more arousing and that the face valence is affected in the direction of the odor valence (Syrjänen et al., 2018, 2019). However, results are not entirely consistent. Using Bayesian statistics, Syrjänen et al. (2017) found evidence against odor effects in face evaluations.

Ratings are also used to study valence congruency effects. One study showed congruency effects between facial expressions and odor valence; faces displaying happiness were rated as more pleasant during exposure to a pleasant odor, and disgusted faces were rated as more unpleasant during exposure to unpleasant odor (Cook et al., 2017). Odors may also affect facial attractiveness, a construct closely related to pleasantness; in one study, faces were rated as younger and more attractive-looking in a pleasant odor context (Seubert et al., 2014). In a similar vein, Dematte et al. (2007) found that faces were rated as less attractive in the presence of an unpleasant odor. However, Cann and Ross (1989) found no effects of pleasant and unpleasant odors on attractiveness evaluations in a blocked design. Finally, one study indicated that such facial evaluations were affected by odor exposure at a subthreshold level. Here, W. Li et al. (2007) found that only participants unaware of the presence of unpleasant odor were influenced to rate neutral faces as less likable. However, the evidence reviewed earlier suggests that also readily perceived odors may have similar influences on face evaluations.

### Olfactory Conditioning: Learned Odor-Face Associations

In conditioning paradigms, associations between stimuli are created by simultaneous presentations. Here, we focus on olfactory conditioning studies that use face and odor stimuli. In studies on this topic, an unpleasant odor (unconditioned stimulus [UCS]) is usually associated with a face conditioned stimulus (CS+) by presenting these concurrently; faces that are not associated with the UCS (CS−) are used as a control. Some studies have used odors as a contextual cue and instead used startle sounds (sudden loud noises) as UCS (Hermann et al., 2000). These designs allow studying how learned cross-modal associations shape the processing of face stimuli; faces associated with unpleasant odors as CS+ may be processed differently from the CS− associated faces. Two studies have shown response-time effects in a gender classification task, but results are inconsistent; CS+ faces associated with an unpleasant olfactory UCS were initially faster at the beginning of the task (Gottfried & Dolan, 2004). However, in another similar study (Gottfried et al., 2002), results showed slowed responses to the CS+ faces conditioned with either pleasant or unpleasant UCS odors. More research with high-powered samples is needed to establish how valenced odor conditioning might modulate face classifications.

Several conditioning studies have shown similar results as when odors and faces are presented concurrently, faces previously conditioned by an unpleasant odor are evaluated more negatively (Hermann et al., 2000; Steinberg et al., 2012, 2013). Steinberg et al. (2013) found that faces conditioned with pleasant odors were preferred over faces paired with clean air and an unpleasant odor. In four experiments, Todrank et al. (1995) found that neutral faces associated with pleasant and unpleasant odors were liked and disliked, respectively. However, this effect was only consistent with odors that were people-related (e.g., soap or sweat like odors) but not for object-related odors such as woody odors. One study showed that faces were rated as more arousing if they were paired with either pleasant or unpleasant odors (Hermann et al., 2000). Thus, similarly to contextual odors, odor conditioning seems to have robust effects on facial evaluation, with odors biasing the evaluation of faces in the odor valence direction. This capacity is highlighted in a study by Steinberg et al. (2012). The authors showed that in a stimulus set of 208 faces, only two CS+ pairings (neutral faces) with the unpleasant UCS odor were enough to affect facial evaluations negatively. We now know that animals can associate aversive odors with visual objects in a fast and flexible manner (Handler et al., 2019). These findings show a similar capacity in humans to rapidly associate faces with aversive odors.

### Z-Curve Results

Using *z*-curve, we found that 75% of the focal tests that were reported were statistically significant. However, if we assume that publication hinges on statistically significant results, 75% might be an overestimate of the true hypothesis confirmation rate. Because many of the studies reported several statistical tests, resulting in a higher chance of obtaining a statistically significant result. To estimate an unbiased ODR, we sampled one test from each study. The results were similar compared with the full sample of *p*-values, with a mean of 77% significant results. The results of the *z*-curve analysis indicated clear evidence of publication bias in the studies in this review. Given the estimated power in the included studies, only 10% of the results were expected to result in statistically significant findings. This discrepancy between the ODR and the EDR resulted in an expected replicability rate of only 47%. That is, if these studies would be exactly replicated, only 47% of the studies would yield similar findings. In the following, we highlight outstanding questions for future research studies and suggest measures that could improve their reliability.

### Future Directions

In the review mentioned earlier, we have highlighted key results emerging from the literature on odor effects on face perception. Several avenues remain to be explored in further research. For example, only a few studies investigated how interindividual variation in odor sensitivity affected odor effects in facial perception. Individuals vary considerably in their odor preferences, which might influence how different odors affect facial perception. For example, people vary in disgust sensitivity (Tybur et al., 2018), which might be especially relevant for how unpleasant odors might affect perception of facial expressions. Here, a newly developed Body Odor Disgust Scale might be a suitable instrument for investigating individual differences in olfactory related disgust (Liuzza, Lindholm, et al., 2017). Although the Body Odor Disgust Scale was developed for measuring body odor-related disgust, it has excellent construct validity in relation to existing disgust scales and has been shown to predict disgust responses to human sweat bio-samples (Liuzza, Olofsson, et al., 2017).

Nevertheless, individual variation is not only relevant for unpleasant odors. For example, we have observed that odors typically regarded as pleasant (e.g., lilac) might be perceived as unpleasant at higher concentrations by some participants (Syrjänen, 2020; Syrjänen et al., 2018). Conversely, an unpleasant odor such as valeric acid is by some participants rated as positively valenced (see Figure 2 for a summary of valence ratings for these odors at three concentrations; data from Syrjänen et al., 2018). Although olfactory valence is sometimes thought of as a hard-wired response, perhaps universal (Yeshurun & Sobel, 2010), our observations suggest this assumption may lead to an underestimation of the individual variation in olfactory valence evaluations. Future work could instead use different odors for each participant to maximize valence effects.

Among the reviewed studies, many have used unpleasant, presumably disgusting odors, and facial expressions such as angry or fearful that do not have a natural connection to these odors. This usage might reflect the fact that anger and fear are well investigated in affect perception research but that these emotions are not easily mapped onto the affective space of odors. Instead, we argue that the basic emotion most well-matched by unpleasant odors is disgust, and for pleasant odors, happiness (Bensafi, Rouby, et al., 2002; Croy et al., 2011; Glass et al., 2014). Future research should consider odors congruent with distinct emotional expressions, and this olfactory-visual congruency should be empirically validated. For example, odors such as smoke may elicit fear in specific contexts (Stevenson, 2010). Thus, the odor might modulate the processing of fearful expressions in a context-dependent way. Future studies might also explore the connection between odors and mood and how these relate to the perception of emotional expressions. Also, interindividual factors such as gender, age, and culture should be investigated in future research. These factors typically affect the perception of emotional information in other sensory modalities such as vision and hearing (Cortes et al., 2017, 2019) and therefore probably also in the context of different odors.

It is important to note that the studies reviewed here are typically not characterized by open research practices such as transparent and preregistered study protocols and open data. In recent years, the low reproducibility of research findings has led to a discussion about methodological improvement in psychological science (Open Science Collaboration, 2015). Transparent and open study protocols might reduce practices that result in nonreplicable findings, such as hypothesizing after results are known (HARKing; Kerr, 1998). Considering that at least half of the studies utilized small sample sizes and that odor effects typically may be assumed to be subtle, we can expect that some of the findings in this area are false positives or negatives (Button et al., 2013). Almost all studies found some effects of odor on facial perception. If we assume that many studies in the field are underpowered, there might be a file drawer problem of conducted but not published studies due to nonsignificant findings. These concerns were confirmed by the *z*-curve analysis. Given the power in the included studies, the EDR was only 10%. Furthermore, the expected replicability rate indicated that 47% of the results would replicate in exact replication studies. The research in this area could also benefit from standardized measures, testing procedures, and stimuli. Practices that have been highly successful in emotion research include using standardized stimuli such as the international affective picture set (Lang et al., 2008) and assessments such as the self-assessment manikin (Bradley & Lang, 1994). Standardization would benefit the research community by making it easier to compare results across studies and sustain a cumulative progression of research.

Because of the relatively small number of published studies to date, open data would be beneficial for the whole community of researchers. Open data practices will enable reinterpretation and creation of novel hypotheses using existing data, and such practices will reduce the need for duplications. On a positive note, several large multinational collaborations are formed to better assess the reliability and generalizability of research findings. In the olfactory research community, the Global Consortium for Chemosensory Research (www.gcchemosensr.org) studies olfactory-related changes in COVID-19 infection (Parma et al., 2020). The first publication from the Psychological Science Accelerator, another large-scale research collaboration, assessed how faces are evaluated in different world regions (Jones et al., 2021). Such efforts might be used as templates for conducting large-scale replication efforts.

## Conclusions

We reviewed studies that span three decades of olfactory research on odor effects on facial perception. Generally, the results indicate that facial expressions are classified more rapidly in the context of odors. Apart from a few studies that show face-odor valence congruency effects, the most consistent finding is that valenced odors affect perceived face valence overall (e.g., faces are perceived as more unpleasant in an unpleasant odor condition). Rapid classification of faces is often unaffected by the congruency of face-odor pairings. However, there is some evidence that an odor’s presence improves the response speed due to overall arousal effects. Establishing important individual differences markers and how odor effects may accumulate as a function of exposure time remain questions of importance for future work. More work on standardizing stimuli and experimental protocols and adopting open research practices may increase the robustness of results and might help address outstanding questions in this field.
